# Transcription Profiling of Malaria-Naïve and Semi-immune Colombian Volunteers in a *Plasmodium vivax* Sporozoite Challenge

**DOI:** 10.1371/journal.pntd.0003978

**Published:** 2015-08-05

**Authors:** Monica L. Rojas-Peña, Andres Vallejo, Sócrates Herrera, Greg Gibson, Myriam Arévalo-Herrera

**Affiliations:** 1 Center for Integrative Genomics, School of Biology, Georgia Institute of Technology, Atlanta, Georgia, United States of America; 2 Caucaseco Scientific Research Center (CSRC), Cali, Colombia; 3 School of Health, Universidad del Valle, Cali, Colombia; New York University School of Medicine, UNITED STATES

## Abstract

**Background:**

Continued exposure to malaria-causing parasites in endemic regions of malaria induces significant levels of acquired immunity in adult individuals. A better understanding of the transcriptional basis for this acquired immunological response may provide insight into how the immune system can be boosted during vaccination, and into why infected individuals differ in symptomology.

**Methodology/Principal Findings:**

Peripheral blood gene expression profiles of 9 semi-immune volunteers from a *Plasmodium vivax* malaria prevalent region (Buenaventura, Colombia) were compared to those of 7 naïve individuals from a region with no reported transmission of malaria (Cali, Colombia) after a controlled infection mosquito bite challenge with *P*. *vivax*. A Fluidigm nanoscale quantitative RT-PCR array was used to survey altered expression of 96 blood informative transcripts at 7 timepoints after controlled infection, and RNASeq was used to contrast pre-infection and early parasitemia timepoints. There was no evidence for transcriptional changes prior to the appearance of blood stage parasites at day 12 or 13, at which time there was a strong interferon response and, unexpectedly, down-regulation of transcripts related to inflammation and innate immunity. This differential expression was confirmed with RNASeq, which also suggested perturbations of aspects of T cell function and erythropoiesis. Despite differences in clinical symptoms between the semi-immune and malaria naïve individuals, only subtle differences in their transcriptomes were observed, although 175 genes showed significantly greater induction or repression in the naïve volunteers from Cali.

**Conclusion/Significance:**

Gene expression profiling of whole blood reveals the type and duration of the immune response to *P*. *vivax* infection, and highlights a subset of genes that may mediate adaptive immunity.

## Author Summary

*Plasmodium vivax* malaria is a debilitating, occasionally life-threatening, and economically burdensome disease in Central Latin America, where 70%- 80% of the population lives with the risk of infection. We performed a gene expression profiling experiment taking advantage of a previously described sporozoite challenge experiment in Cali, Colombia that reported more severe malaria symptoms in subjects who have never experienced malaria. We show that no major differences are seen in the transcriptomes of uninfected naïve and semi-immune volunteers prior to infection, but differential expression of both neutrophil and interferon-related genes was evident at onset of malaria. Several hundred genes showed a stronger response in the naïve individuals just as parasites appear in the peripheral blood, and these fall into several pathways of interest. These findings show how information from gene expression profiling of whole blood can reveal the type and duration of the immune response to *P*. *vivax* infection, and highlights a subset of genes that may mediate adaptive immunity in chronically exposed individuals.

## Introduction

One of the features of *Plasmodium* species that make them such pernicious parasites is their ability to avoid the host immune system [1,2]. While this is achieved in part by virtue of their complex life cycle that includes intra-erythrocyte cycling and periodic sequestration in various tissue compartments [2], it is also clear that *Plasmodium* infection causes short- and probably long-term modification of host immune function. Molecular methods are shedding some light on the mechanisms behind these modifications. For example, it is now clear that exposed individuals generally do mount an antigen response to *Plasmodium* antigens that persists [3,4], and that several biochemical pathways are engaged, including interferon and cytokine signaling, membrane lipid modification, and reactive oxygen species metabolism [5]. Host factors including genetic variation, both within and between populations, play a role in modulating immunity in malaria, as does the microbiome [6–9].

An important factor influencing the clinical course of disease is prior exposure to malaria. Adults and older children tend to experience reduced prevalence of malaria infection and have less severe symptoms [10,11]. Nevertheless the mechanisms responsible for host resistance to malaria are still poorly understood. As a prelude to evaluation of vaccine efficacy in a Colombian population, we recently carried out a challenge experiment in which we described the responses of immunologically naïve and semi-immune individuals to deliberate infection with *Plasmodium vivax* through mosquito bites [12]. All nine volunteers from a malaria endemic region near the town of Buenaventura were weakly positive for IgG antibodies to sporozoites or blood stage proteins prior to the experiment, and after challenge eight of them showed increased antibody titers against blood stages. Similarly, five of seven naïve volunteers from the city of Cali converted to sero-positivity that was generally maintained for at least four months. While there was no significant difference in the time to first appearance of blood stage parasite assessed by thick blood smears (12 to 13 days in both groups) or by polymerase chain reaction (PCR) (around 9 days), the naïve volunteers experienced classical early malaria symptoms, whereas the semi-immune volunteers were for the most part nearly asymptomatic, at least at the day of diagnosis when curative prophylaxis was administered [12].

In order to begin to characterize the molecular basis for this difference in clinical course of disease as a function of prior exposure to malaria, we report here two types of transcriptome profiling of peripheral blood samples from the Colombian challenge experiment volunteers. First we used targeted measurement of a set of 96 highly informative transcripts by nanoscale Real Time PCR (RT-qPCR) [13] in order to generate a time course of the infection transcriptional response. Second, we used RNASeq [14] on a subset of six volunteers contrasting baseline and incident malaria, to ask whether (i) there is a difference in immune profiles between naïve and semi-immune individuals in the absence of infection, and (ii) patent infection results in a differential transcriptional response that may hint at the molecular basis of long-term immunity. We also contrasted our findings with those of cross-sectional studies, concluding that history of exposure is just one of many factors mediating host–parasite interactions in malaria.

## Methods

### Experimental design and ethics statement

The experimental design protocol of this research was approved by the Institutional Review Boards (IRB) at the Malaria Vaccine and Drug Development Center (CECIV, Cali) and Centro Médico Imbanaco (Cali). Volunteers were adults and were extensively informed about the risks of participation. Before signing the written consent, all volunteers had to pass an oral or written exam related to the trial and its risks. Clinical trial was registered under registry number NCT01585077. It is described in more detail in Arévalo-Herrera et al. [12], which reports the clinical responses to malaria challenge. Sixteen Duffy-positive (Fy+) male and female volunteers (9 semi-immune, previously exposed to malaria, from Buenaventura and 7 immunologically naïve with respect to malaria, from Cali; 10 men and 6 women) were enrolled.

**Fig 1:**
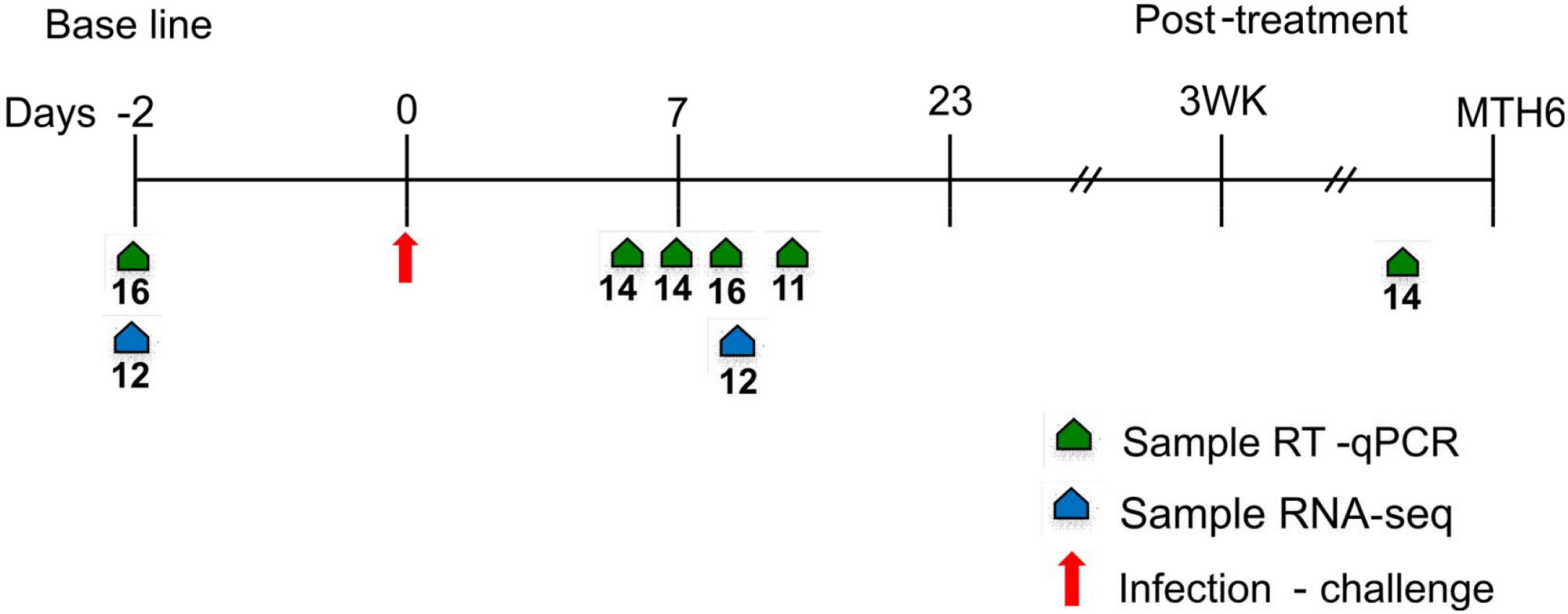
Experimental design. The time line sample collection, 85 total samples for RT-qPCR analysis and 24 total samples for RNASeq. Green arrows represent timepoints where RT-qPCR Pre-challenge, 16 samples (7 Cali, 9 Buenaventura); Day 5, 14 samples (6 Cali, 8 Buenaventura); Day 7, 14 samples (5 Cali, 9 Buenaventura); Day 9, 16 (7 Cali, 9 Buenaventura); Diagnosis by tick blood smear day (Day 12–13), 11 samples (5 Cali, 6 Buenaventura) and Month 4, 15 samples (6 Cali, 9 Buenaventura). Blue arrows shows samples used for the RNASeq analysis 12 per each timepoint Diagnosis day and Pre-challenge (6 Cali, 6 Buenaventura) 24 total.

Volunteers where invited to the vaccine center two days (day -2) prior the challenge day (day 0) for physical examination and blood sample collection. Fig 1 summarizes the blood sampling strategy. Blood samples used for the RT-qPCR experiment were collected on day -2 (pre-challenge), day 5, day 7, day 9, on the day of first detection of *Plasmodium* by thick smear test (day 12–13), and on month 4. RNASeq analysis, also approved by the Georgia Tech IRB, was performed for 12 individuals (six each from Buenaventura and Cali) for two of the timepoints, namely the diagnosis day and baseline (pre-challenge day).

For each sample, approximately 1 mL of blood in 2 mL of buffer was collected into a Tempus tube, which preserves whole blood RNA at 4°C indefinitely. Whole blood mRNA was extracted using Tempus Blood RNA Tube isolation kits provided by the manufacturer Applied Biosystems, and the sample quality was determined based on the Agilent Bioanalyzer 2100 RNA Integrity score (RIN). Two samples had RIN approximatley 4 but these were not outliers in the analysis and there was no indication that RNA degradation influenced the results meaningfully.

### RT-qPCR

Reverse Transcription followed by quantitative PCR (RT-qPCR) was performed using Fluidigm 96×96 nanofluidic arrays targeting a set of 96 transcripts that are broadly informative of the major axes of variation for peripheral blood gene expression from Preininger et al. [15] at six timepoints (Pre-challenge, day 5, day 7, day 9, Diagnosis (Dx) and month 4). The RT-qPCR was completed in three steps: (1) Total whole blood RNA was converted to single stranded cDNA using polyT priming of reverse transcription, (2) the 96 targeted genes were pre-amplified in a single 13-cycle PCR reaction for each sample following conditions outlined in the manufacturer’s protocol by combining cDNA with the pooled primers and EvaGreen Mastermix (Fluidigm BioMark), and (3) qPCR reactions were performed for each sample and individual gene on each sample on a 96×96 array with 30 amplification cycles. Average Ct value was calculated at a point in which every reaction is in the exponential phase to ensure accuracy and precision of amplification. In order to make the analysis more easily comparable with traditional transcript abundance measures such as those obtained with microarrays or RNASeq, each Ct value was subtracted from 30, setting missing values to 0. Since small Ct values correspond to high transcript abundance, this subtraction yields values ranging from 0 (no expression) to 30 (very high abundance). All measurements are reported in S1 Table.

### RNASeq

Library preparation for RNASeq was performed using the Illumina TruSeq Low Throughput (LT) RNA Sample Preparation Protocol. Short read sequencing was performed in rapid run mode with eight samples per lane on an Illumina HiSeq 2500, generating 100 bp paired-end libraries with an average of 15 million paired reads per sample, and then sequencing on an Illumina HiSeq2100 at Georgia Institute of Technology.

The raw RNASeq reads (Fastq files) for each sample were tested using FastQC software analysis to check the quality of the data (S2 Table) and then aligned to the reference human genome (hg19 / GRCh37 assembly) using Bowtie as the short read aligner, and splice junctions were identified using TopHat2 in the Tuxedo protocol [16]. After alignment, estimation of transcript abundance measures as fragments per kilobase of exon per million aligned fragments (FPKM) values was performed using Cufflinks [16]. As a quality control for high variance associated with low abundance, genes with FPKM greater than 2.5 averaged across the 24 samples were retained for downstream analyses, representing 6,154 genes.

FPKM values were then transformed to logarithm base 2 to guarantee that the data were more normally distributed and to simplify the interpretation of the scale of differential expression (each unit difference corresponds to a two-fold difference in abundance). The supervised normalization of microarray (SNM) procedure was then used to normalize the data with the R package from Bioconductor [17], fitting location and timepoint as the biological variables, and individual as the adjustment variable (fit but not removed). All downstream analyses were performed on this normalized data set provided as S3 Table.

### Blood Informative Transcripts (BIT)

Many gene expression profiling experiments start with analysis of Principal Components, but since these are study specific, we also performed an analysis focused on large sets of genes that have been found to consistently covary in peripheral blood, namely blood informative transcript (BIT) axes analysis [15]. This analysis focuses on 9 common axes of variation that we detected in all human peripheral blood gene expression datasets that we have examined. They are related to 28 modules of co-expressed genes described by Chaussabel and colleagues [18] and found to be dysregulated in various immune diseases, but which collapse into 9 larger Axes of transcripts that covary in healthy adults. Each axis includes from several hundred to several thousand genes that gene set enrichment analysis suggests are involved in particular immune functions, broadly speaking, T cell signaling (Axis 1), reticulocyte number (Axis 2), B cell signaling (Axis 3), and inflammation (Axis 5) or specific immune or physiological responses (Interferon signaling, Axis 7).

Blood informative transcript (BIT) axes analysis was performed by generating the first PC for the 10 genes that are most strongly correlated with each of the 9 Axes reported in [15]. Principal component one (PC1) for each of these 10 sets of BIT provide a summary axis score, to which all of the other genes in the Axis positively correlate, nominally with a Pearson correlation coefficient greater than 0.5 as listed in S4 Table. This Axis score is then contrasted with respect to the covariates of interest (primarily time-point, location, and the interaction between them, but in exploratory analyses gender, parasitemia, and individual) using standard parametric t-tests or analysis of variance, or with linear regression.

### Statistical analyses

Most statistical analyses of both the Fluidigm and RNASeq datasets were performed in JMP Genomics version 5 (SAS Institute, NC), starting with the Basic Expression Workflow, which performs principal components analysis (PCA), and computes a weighted total contribution of the covariates of interest to the axes (principal variance components analysis, PVCA). Linear regression was then used to assess the relationship between the individual covariates and PC, and/or analysis of variance was used to detect differential expression between locations or timepoints. We fit models with timepoint and location as fixed effects, and with individual as a random effect, in order to control for the effect of differential responses among individuals within each sample. No differences in the significance of the fixed effects were observed compared to models without individual, implying that this source of variation is minimal, and we report the significance of the full model.

A Benjamini-Hochberg 5% false discovery rate was used to select differentially expressed genes. Volcano plots contrast the significance (negative log10 of the p-value, NLP) against the fold difference (normalized log2 Ct or FPKM units) between specific conditions. Hierarchical clustering was performed using Ward’s method, and approximately unbiased boostrap support AU values were computed with the R program pvclust [19].

For the RNASeq there were 6 individuals of each gender, but they were asymmetric with respect to location, since 5 females were from Buenaventura and 5 males from Cali. Gender did not however account for a significant proportion of the major PC of gene expression. To confirm this, we fit models with gender, time and location for each Axis, and in each case the gender term was non-significant and the other terms were unaffected.

### Comparison with malarial gene expression in a study from Benin, West Africa

In order to identify whether location influences the axes of variation in another study, we reanalyzed data from Idaghdour et al. [9] who characterized whole blood transcriptomes of infants from the West African Republic of Benin, infected with *Plasmodium falciparum*. They reported on 61 healthy controls from a hospital in the city of Cotonou, and 92 cases drawn approximately equally and without bias with respect to parasitemia levels from Cotonou and the village of Zinvié, located 36 km from Cotonou (GEO accession number GSE34404). They identified parasitemia as the major factor influencing transcript abundance overall, but also described a location effect that is considered with respect to the BIT axes here.

### Accession numbers

The RNASeq dataset has been deposited into the Gene Expression Omnibus archive (GEO) under accession number GSE67184 and RT-qPCR data accession number GSE67470.

## Results

### RT-qPCR comparison of naïve and semi-immune responses to infection

The first objective of this study was to compare the time course of transcriptional changes during response to infection, between naïve and semi-immune volunteers. There were 16 volunteers in all, 7 from Cali who had not previously been exposed to malaria, and 9 from Buenaventura, a village in an endemic region for the disease, all of whom had experienced between 2 and 5 mild bouts of malaria. Fig 1 summarizes the peripheral blood sampling scheme from 14 volunteers at Day 5 following exposure and again at Day 7, from 16 volunteers at Day 9 when PCR later confirmed initial appearance of blood-stage parasites, from 11 volunteers on Days 12 or 13 when parasitemia was diagnosed in thick blood smears, and from 14 volunteers four months after the initiation of the experiment. As mentioned above, there were no significant differences between the two groups either in the length of the pre-patent period or the level of parasitemia attained before administration of a curative cocktail of anti-malarial drugs.

**Fig 2:**
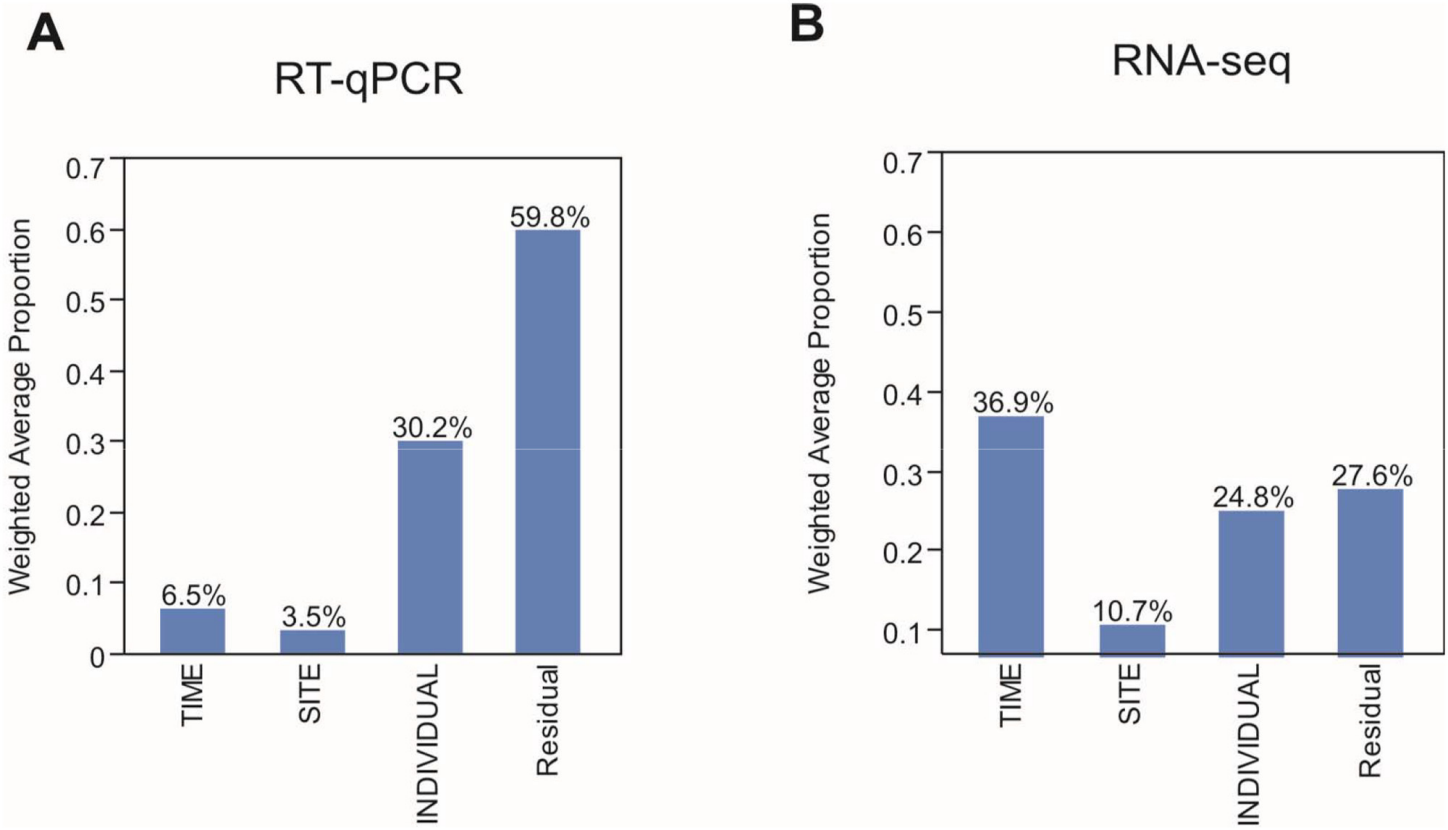
Principal component variance component analyses. Bar graphs shows the weighted average of the variance captured by the first five principal components among samples that is explained by Time (PRE, DAY5, DAY7, DAY9, Diagnosis, MTH4), Site (Cali, Buenaventura) and individual, indicating that most the variability is among individual for RT-qPCR (A) and Time (PRE and Diagnosis day) for RNASeq data set (B).

Whole blood gene expression was monitored in each of the 85 samples using a Fluidigm nanoscale RT-qPCR array targeting 96 genes referred as “blood informative transcripts” (BIT) (S5 Table). These BIT consistently capture the covariance of over half of the genes expressed in blood, specifically serving as biomarkers for 10 conserved axes of variation. Across all of the gene expression measurements, 30% of the variance was among individuals, and just 6.5% between the timepoints, with very little differentiation between the naïve and pre-immune volunteers (Fig 2). The remainder of the variance was due to random biological or technical noise, or to the covariance of gene expression along the Axes.

**Fig 3:**
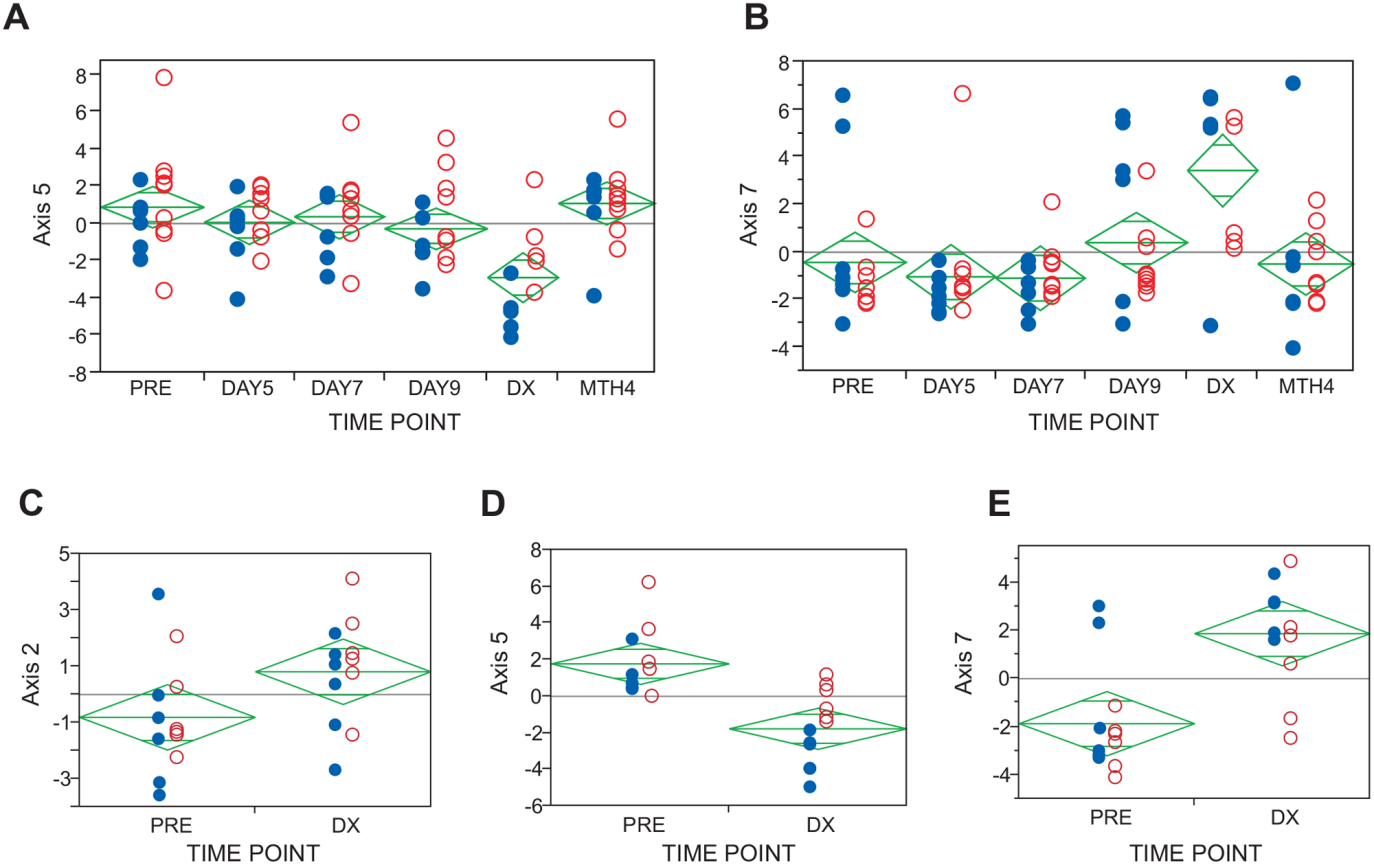
Axis of variance analysis. Each plot shows the differences in Axis scores at 6 different timepoints for RT-qPCR (**A and B**) and two for RNASeq **(C-E**); Blue solid point represents Cali, and red open circles represent Buenaventura. P-values for the effect of timepoint including location as a fixed effect and individual as a random effect in the RNASeq data are 0.016 (Axis 2), 4.4×10^-5^ (Axis 5), 0.0003 (Axis 7).

We confirmed that most of the genes were co-regulated in this dataset by observing a strong correlation of expression for each of the 10 BIT for each Axis, and then generated Axis scores as the first principal component of the variance of those 10 BIT. Only two of the Axes were differentially expressed among timepoints in the Fluidigm data (Fig 3A and 3B). Axis 5 is related to innate immune signaling and neutrophil number, and seems to decline at Diagnosis, surprisingly, implying a mild reduction in inflammatory gene activity. Axis 7 represents Type 1 interferon induction and is, as expected, elevated at diagnosis, reflecting a transient specific immune response. Both axes had returned to close to baseline levels three months after recovery. No other gene expression differences detected by this targeted RT-qPCR analysis were associated with time or population. These results are consistent with previously observed stable maintenance of peripheral blood gene expression profiles in healthy adults. A caveat to this analysis is that it is possible that other genes not included in the targeted set of probes do change in expression prior to the diagnosis of parasitemia, or alternatively do not return to baseline after recovery.

### RNASeq comparison of naïve and semi-immune responses at time of parasitemia

In order to obtain a more comprehensive picture of the changes in gene expression as parasites first appear in the blood, we performed RNASeq on 6 volunteers each from Cali and Buenaventura, both at Baseline and Diagnosis. An average of 15 million paired-end 100bp short read alignments to the human reference genome were obtained for each sample, allowing us to estimate transcript abundance for each of 6,154 genes. Analysis of variance was used to contrast gene expression relative to population and timepoint, and to assess the interaction between these two factors. Fig 2B shows that 25% of the total variance was among individuals, similar to the Fluidigm observation, and that very little differentiation was seen between populations. However, just over one third of the variance was between Baseline and Diagnosis samples, implying a much greater response to infection than suggested by the RT-qPCR data, though it should be noted that when only contrasting the two most different timepoints, this contrast was expected to account for more of the variance.

The differential expression of Axes 5 and 7 was confirmed by the RNASeq data (Fig 3D and 3E), which also suggested divergence of Axis 2 (Fig 3C). Individual variability in response was minimal, as inclusion of individual as a random effect in the models had no effect on the proportion of variance due to either the time-of-diagnosis or population sampled. Up-regulation of Axis 2 is likely to be a sign of elevated erythropoiesis since they are enriched for erythrocyte-related function [15] and reanalysis of the dataset reported by Whitney et al. (2003) [20] shows that the genes in this axis are highly correlated with reticuloycte count, suggesting a mild physiological response to loss of red blood cell function even in the early stages of malaria. Interestingly, the increased resolution of RNASeq suggests differential responses of Axes 5 and 7 between the naïve and semi-immune populations. Specifically, the neutrophil and TLR-signaling associated with Axis 5 is much weaker in the naïve individuals (Fig 3D, solid blue points, p = 0.0008, though the location×timepoint interaction term is not significant, p = 0.13), whereas the induction of interferon signaling is variable in semi-immune volunteers (Fig 3D, open red circles), two of whom showed no response. The directional trends were the same in the Fluidigm data, but less apparent.

**Fig 4:**
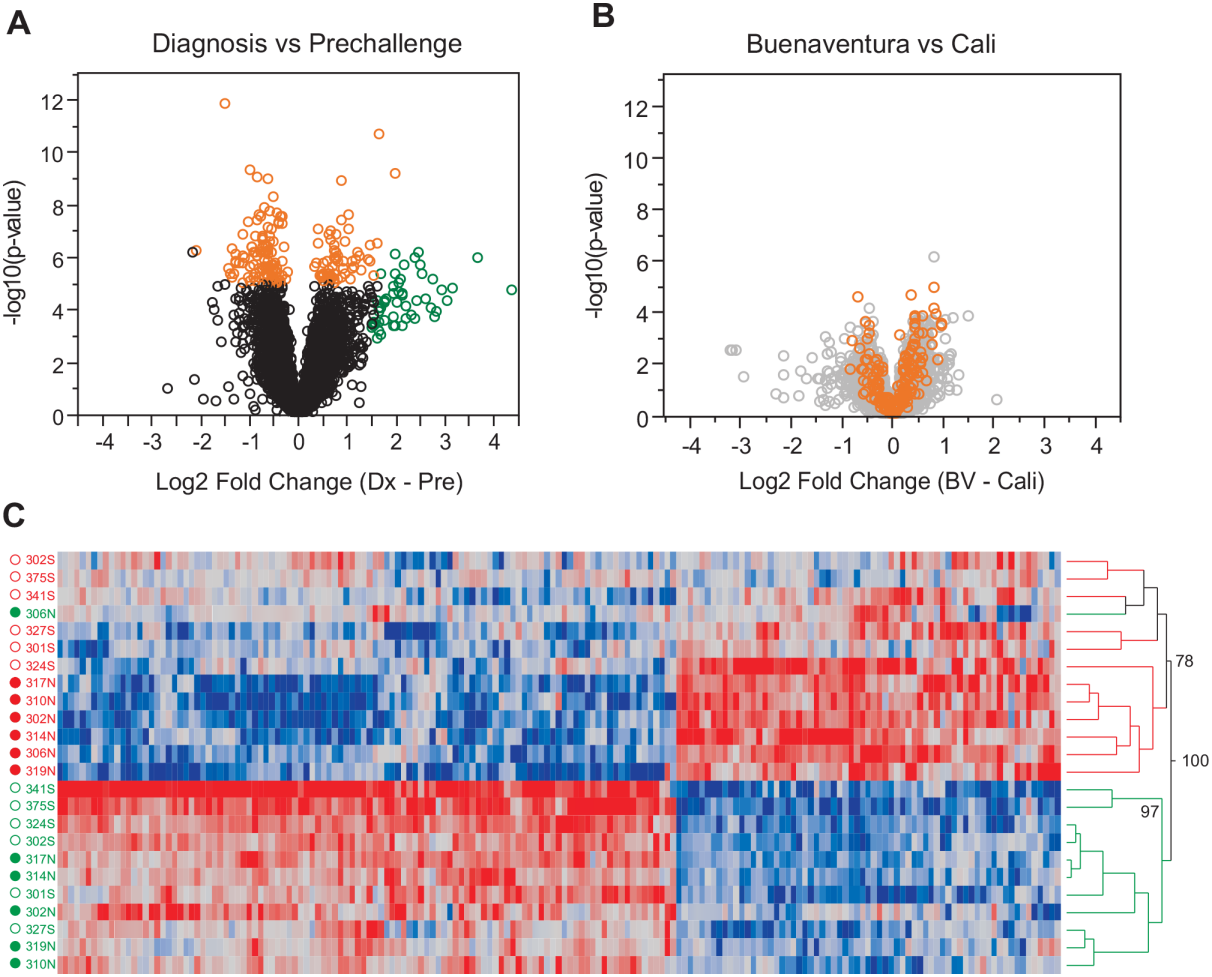
Differential expression in whole-blood RNASeq data set. Volcano plots of statistical significance vs. magnitude of differential expression for the contrasts between timepoint in (A) and by location in (B), highlighting significant genes for the timepoint effect in orange. Y axis shows the significance as–log10 P value, and x-axis shows the magnitude of the difference log2 units (highlighting significant genes at NLP>5 for the timepoint contrast). (C) Heat map showing two-way hierarchical clustering of transcripts (columns) in each sample (rows) of 175 genes that show a significant timepoint-by-location interaction effect at p<0.05. Red cells indicate high expression, blue low, gray intermediate. Green sample labels represent Baseline (Pre-challenge) and red labels represent Diagnosis day, solid points represent Cali (N, naïve), and open circles represent Buenaventura (S, semi-immune). Approximately unbiased bootstrap support (AU) values computed with *pvclust* are indicated beside three deep nodes.

Consistent with timepoint rather than population explaining a large proportion of the variance, gene-specific differential expression analysis revealed more than 250 transcripts up- or down-regulated at the experiment-wide threshold of p<10^-5^ (Fig 4A), but only two transcripts more highly expressed in Buenaventura and none in Cali (Fig 4B). Approximately 50 genes show more than 2-fold up-regulation at Diagnosis relative to Baseline yet are less significant than many of the orange-colored genes (Fig 4A, green-colored genes). The reason is that these genes are even more highly upregulated in a subset of individuals, namely the naïve (Cali) volunteers. In fact, 175 genes show a significant timepoint-by-population interaction effect at p<0.05 (ANOVA, Fig 4C; S6 Table). These are represented in the heat-map in Fig 4C, showing two-way hierarchical clustering of transcripts in samples, two-thirds of the genes are actually down regulated at Diagnosis day (red sample labels, top). Interestingly, there was a marked distinction between the two timepoints (Fig 4C) in the sense that the Baseline samples were intermingled with respect to whether they were from the naïve or semi-immune populations, whereas the Diagnosis ones showed a near-perfect separation with respect to pre-immune exposure (bootstrap support 78%). In other words, most of the genes showing an interaction effect were more strongly up- or down regulated in the naïve than semi-immune individuals. An exception was a Baseline sample from a Cali volunteer (number 306), which clustered with the Diagnosis set but still showed a robust response to malaria infection along with moderate thrombocytopenia and leukopenia, as did Cali 310 who was not an outlier.)

### Nature of the differential response to malaria

**Fig 5:**
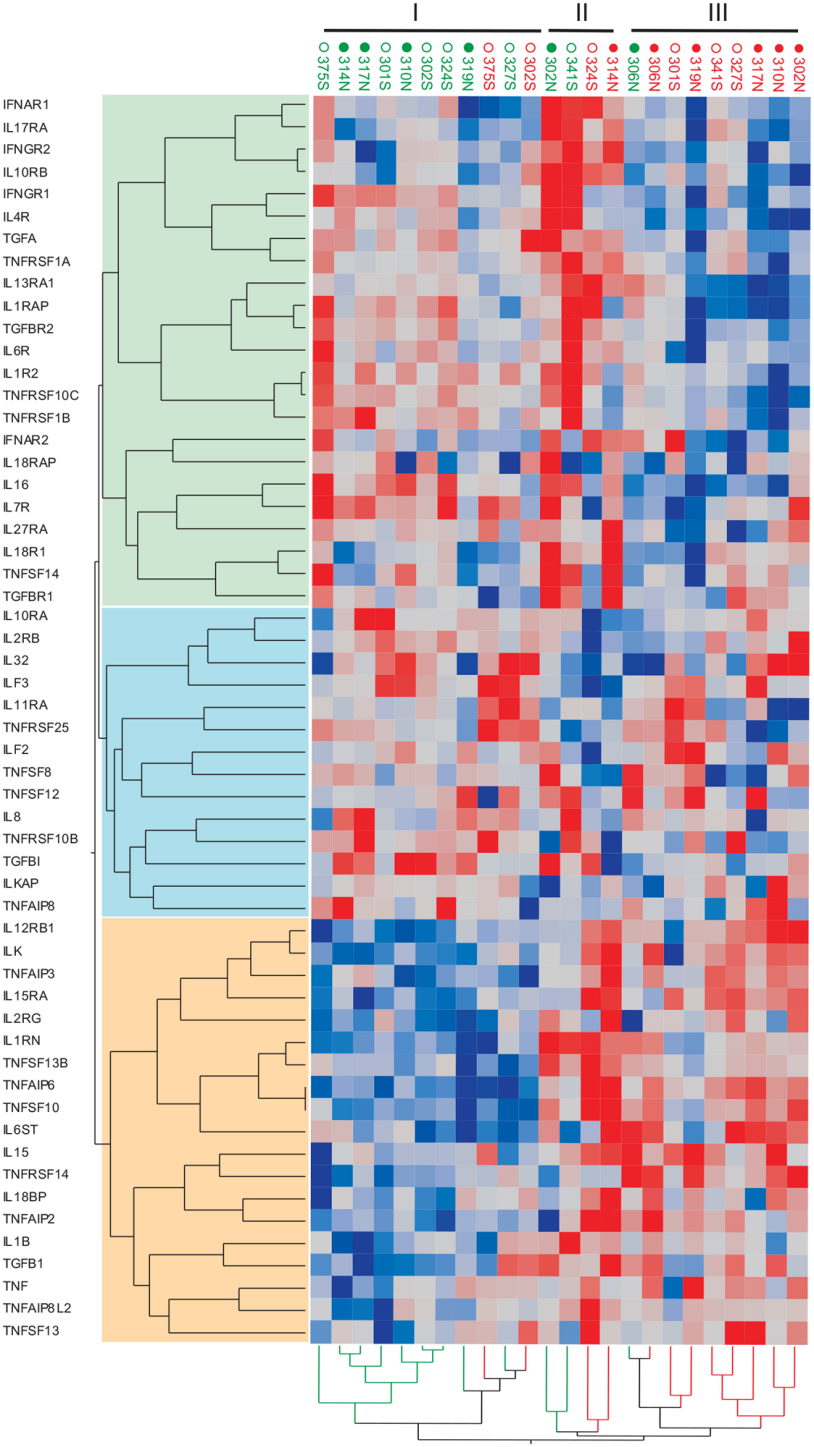
Heat map of Interleukins, Interferon (IFN), Transforming Growth Factor (TGF) and tumor necrosis factor (TNF) genes. All genes with names starting with IL, IFN, TGF or TNF were selected and subject to two-way hierarchical analysis. Red indicates high expression, blue low expression, and gray intermediate. Three groups of samples (I, II and III), and three clusters of genes (green, blue and yellow) are indicated. Baseline (Green labels) and Diagnosis samples (Red labels) are separated, excluding the outlier Cali 306N Baseline sample and 375S and 302S. Solid points represent Cali (N, naïve), and open circles represent Buenaventura (S, semi-immune).

Given the importance of cytokines to regulation of the immune response, we specifically analyzed the expression of many of the genes in the RNASeq dataset that are related to Interleukin (IL), interferon (IFN), tumor necrosis factor (TNF), and transforming growth factor (TGF) signaling. This analysis revealed three groups of samples, and three clusters of genes (Fig 5). Once again, the Baseline and Diagnosis samples were separated, excluding the outlier Cali 306 Baseline sample and two others, but in this case there was no clear separation relative to pre-infection malaria status. One cluster of 14 genes, including IL32 and IL8, was not differentially expressed. Another cluster of 23 genes, including the IL4R, IL6R, and IL7R and IL17R receptors, was upregulated at Baseline, particularly strongly in three volunteers (Cali 302 and Buenaventura 341 and 375). The third cluster of 19 genes, including TNF, IL1B and IL15, showed the opposite tendency, namely up-regulation at Diagnosis, particularly strongly in two samples (314 from Cali and 324 from Buenaventura). These results imply that there is strong co-regulation of the cytokine response and infection, but that this is not mediating the differential response between naïve and semi-immune individuals. This is somewhat surprising, especially given that the experience of fever was significantly different between the two populations, who might have been predicted to differ with respect to the pyrogenic cytokines IL1, IL6, IL8 and TNF.

Closer examination of the differentially expressed genes between Baseline and Diagnosis suggested a complex pattern of cross-regulatory interactions. The up- and down-regulated cytokines for example both include pro- and anti-inflammatory peptides and their receptors. Similarly, there appear to be counter-balancing signal transduction profiles: *JAK1* and *RAF1* are both strongly down-regulated in all volunteers at Diagnosis, whereas IL6ST and SOS1 are up regulated.

**Fig 6:**
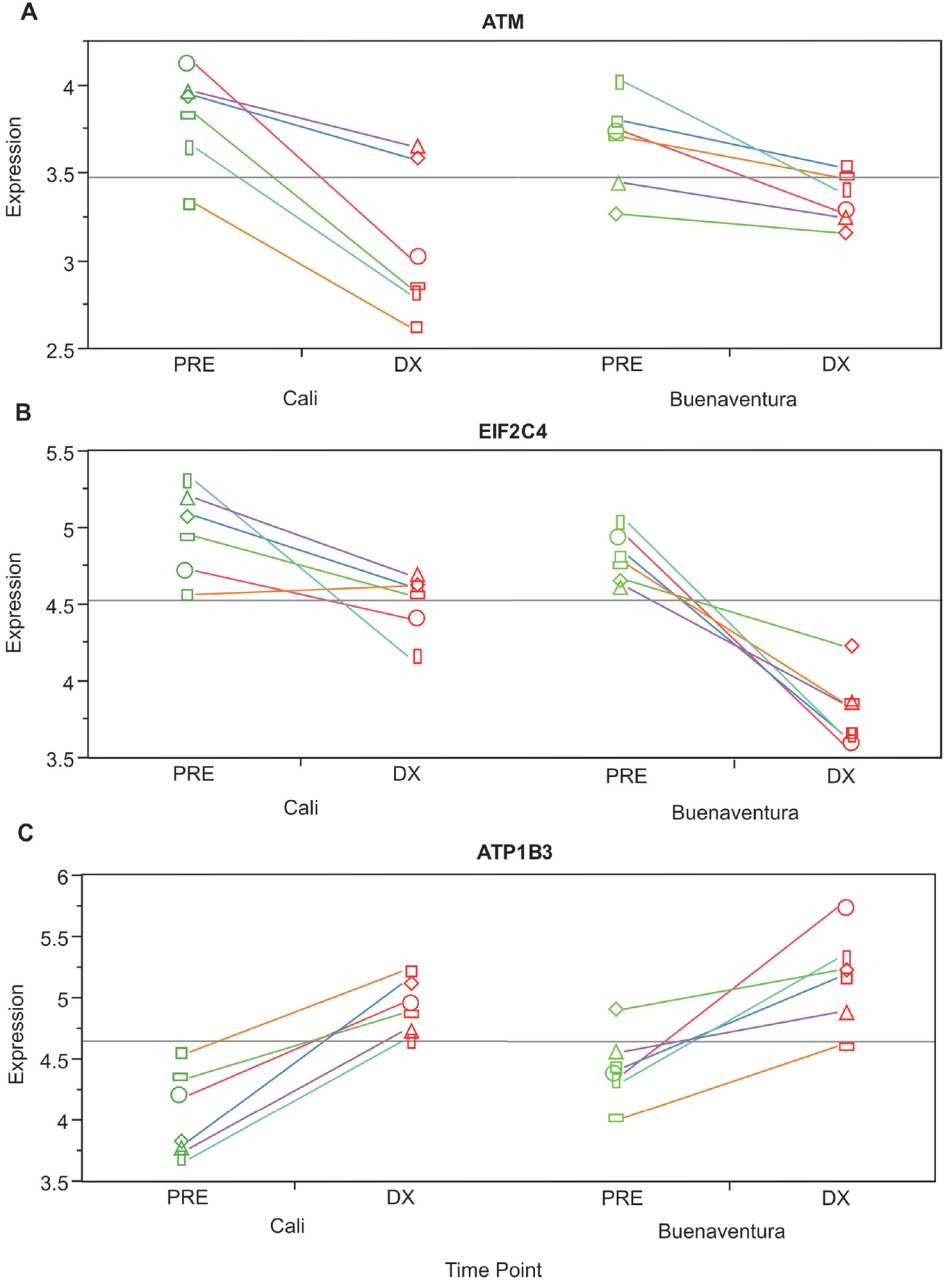
Transcriptional interaction effect between location and timepoint. Examples of the interaction effect showing gene *ATM* up-regulation in Cali (A), gene *EIF2C4* down-regulation in Cali (B) and gene *ATP1B3* with no significant change (C).

Among the 175 genes showing a significant timepoint-by-population interaction effect, namely a stronger response at diagnosis in the immunologically naïve individuals, there are several types of gene functions of interest (Table 1). These include lysosomal components (*CTSH*, *RILP*), regulators of macrophage activity (*CD163*, *MMP25*, *SIRPA*, *TBC1D14*, *TNFSF13*), splicing factors (*EIF2C4*, *SNRPB2*, *SNRPG*), lipid biosynthesis (*DGAT2*, *LPPR2*), solute carriers (*S100P*, *SLC6A6*, *SLC11A1*, *SLC7A7*), signal transduction (*G3BP1*, *GAB3*, *MAPK13*, *TLE3*) and Cell Cycle and DNA damage response (*ATM*, *PRKDC*, *ARID4A*). Some genes with an interaction effect showed stronger down-regulation in Cali (Fig 6A, *ATM*), or stronger down-regulation in Buenaventura (Fig 6B, *EIF2C4*), compared with one that showed a similar up-regulation at both locations (Fig 6C, *ATP1B3*).

**Table 1:** Timepoint-by-population interaction genes at p<0.05 group function.

Gene	p-value	Function
**Immune Regulation**		
*MME*	0.0438	This gene encodes a common acute lymphocytic leukemia antigen that is an important cell surface marker in the diagnosis of human acute lymphocytic leukemia
*CXCR2P1*	0.0036	Interleukin 8 Receptor, Beta Pseudogene—Non annotated
*PECAM1*	0.0278	Cell adhesion molecule that is required for leukocyte transendothelial migration (TEM) under most inflammatory conditions.
*MAFB*	0.0234	Transcriptional activator or repressor. Plays a central role in controlling lineage-specific hematopoiesis repressing ETS1-mediated transcription of erythroid-specific genes in myeloid cells. Is necessary for cell differentiation of monocytic, macrophage, podocyte and islet beta.
**Signal transduction**		
*MAPK13*	0.0182	Is one of the four p38 MAPKs which play a critical role in the cascades of cellular responses induced by extracellular stimuli like physical stress controlling the activation of transcription factors like ELK1 and ATF2 or proinflammatory cytokines.
*GAB3*	0.0092	Is related to numerous growth factor and cytokine signaling pathways.
*G3BP1*	0.0385	Is a heterogeneous nuclear RNA-binding protein and also a constituent of the Ras signal transduction pathway.
*TLE3*	0.0086	Transcriptional co-repressor that binds to a diverse number of transcription factors. Constrains the transcriptional activation, which is facilitated by CTNNB1 and TCF family members in the Wnt signaling.
**Splicing**		
*SNRPG*	0.0065	Plays an important role in the splicing of the cellular pre-mRNAs.
*SNRPB2*	0.0173	Encoded protein might play an important role in pre-mRNA splicing.
*EIF2C4*	0.0184	Members of this argonaute protein family are related to RNA silencing and are evolutionarily conserved.
**Lysosome activity**		
*CTSH*	0.0074	Protein encoded by this gene is a lysosomal cysteine proteinase; this protein plays an important role in the overall deprivation of lysosomal proteins.
*RILP*	0.0166	Related to the regulation of lysosomal morphology and distribution.
**Cell Cycle, DNA Damage Response**		
*ATM*	0.0154	Cell cycle checkpoint kinase. This genes is involved in signal transduction and cell cycle control. May works as a tumor suppressor.
*PRKDC*	0.0028	Sensor for DNA damage.
*ARID4A*	0.0342	Relates with a viral protein-binding domain at the retinoblastoma protein (pRB) this regulates cell propagation.
**Phagocytes**		
*TNFSF13*	0.0086	Plays a part in regulation of tumor cell growing. This gen might be involved in monocyte/macrophage-mediated immunological activities.
*TBC1D14*	0.0101	Adverse regulator of starvation-induced autophagosome formation.
*CD163*	0.0129	This gene is exclusively expressed in monocytes and macrophages. Functions as a severe phase-regulated receptor related to the clearance and endocytosis of hemoglobin/haptoglobin complexes by macrophage.
*SIRPA*	0.0227	Facilitates negative regulation of phagocytosis, mast cell stimulation and dendritic cell activation.
*MMP25*	0.0216	Response to bacterial infection and/or inflammation
**Extracellular sensing**		
*SLC6A6*	0.0337	This gene encodes a multi-pass membrane protein that is a member of a family of sodium and chloride-ion related transporters.
*S100P*	0.032	Might function as calcium sensor and contribute to cellular calcium signaling.
*SLC11A1*	0.0398	The protein work as a divalent change metal (iron and manganese) transporter involved in iron absorption and host resistance to some pathogens.
*SLC7A7*	0.0369	Is a transporter that is found in epithelial cell membranes where it transfers large neutral amino acids from the cell to the extracellular area.
**Lipid Biosynthesis**		
*DGAT2*	0.0375	This gene encodes one of two enzymes which catalyze the final reaction in the synthesis of triglycerides
*LPPR2*	0.012	Activity of phosphatide phosphatase
**Other**		
*HAL*	0.0249	Histidase converts histidine into ammonia and urocanic acid
*TBXAS1*	0.0027	Is an enzyme that plays a role in numerous pathophysiological processes that includes hemostasis, cardiovascular disease, and stroke.
*POGK*	0.0123	Exact function of the protein encoded by this gene is unknown.
*FAM212B*	0.0365	Uncharacterized Protein.
*KIAA0232*	0.0428	Uncharacterized Protein.

### Comparison with effect of parasitemia on gene expression reported from Benin, West Africa

**Fig 7:**
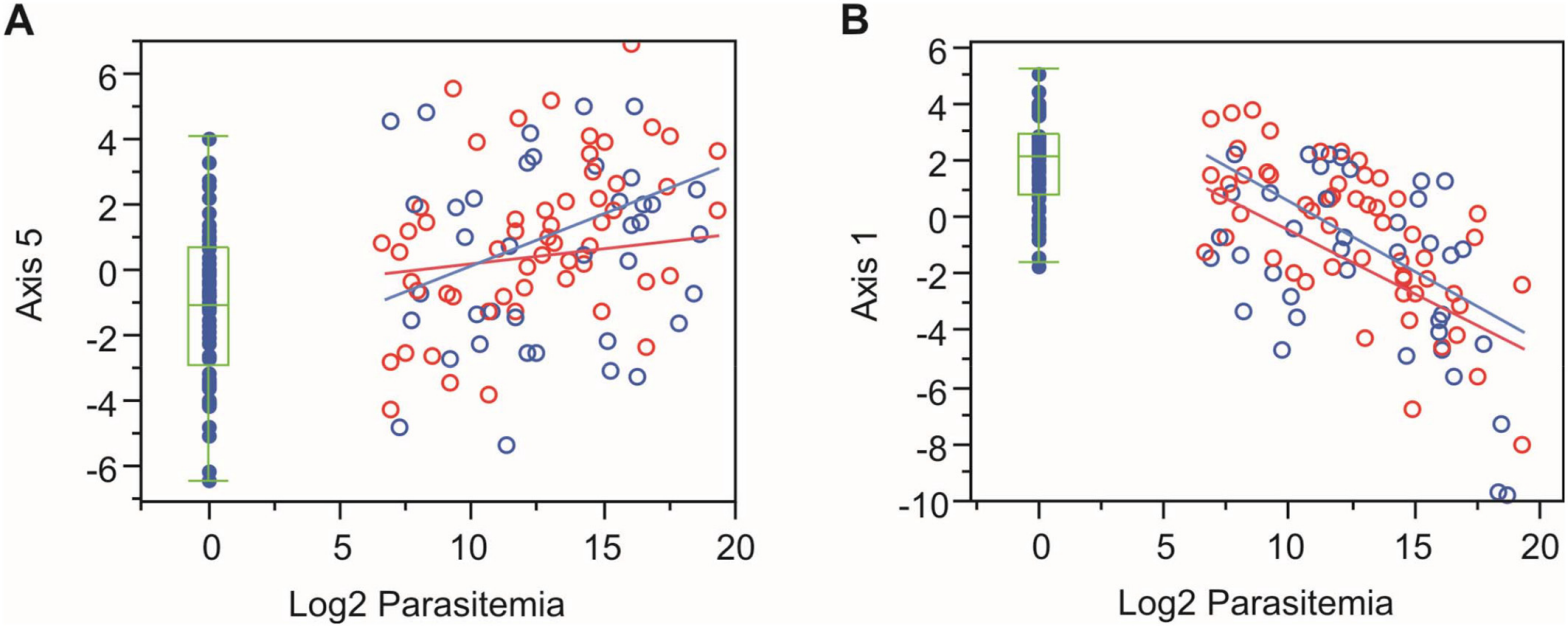
Log2 Parasitemia and Axis 2 and 5 Benin Study, West Africa. Significant correlation is evident between parasitemia and axis of variation, even though unrelated to location. **A.** Axis 5, r = 0.20, p = 0.0079. **B.** Axis 1, r = -0.47, p = 1.4×10^-9^. Neither interaction effect is significant, indicating that the correlation is similar in both locations. Each plot shows the correlation between location and axis of variation, blue represents samples from Cotonou, red represents samples from Zinvié; solid points represent control and open circles represent malaria samples.

Finally, we reanalyzed an infant malarial gene expression dataset from Benin [9]. All samples were collected within a period of 10 weeks in the Spring of 2010, and transcript abundance data was generated on Illumina HumanHT-12 BeadChips for 155 individuals (61 controls from Cotonou, 24 high parasitemia from the village of Zinvie, 52 low parasitemia from Zinvie, and 18 from the city of Cotonou). Critical differences relative to our study include (i) comparison with *P*. *falciparum* rather than with *P*. *vivax* infection, (ii) infants versus young adults comparison, and (iii) cross-sectional rather than Baseline vs Diagnosis analysis. Nevertheless, a significant correlation (Fig 7A and 7B) was observed between parasitemia and two Axes of variation, Axes 1 and 5. However, in this case there was activation of the innate immunity/inflammation genes as parasite burden increases. Axis 1, which is enriched for T-cell signaling activity [15], was strongly reduced as parasitemia increased, but like Axis 5, not significantly affected in the infants with low parasitemia. From 32 genes showing a significant interaction effect between timepoint and population in our challenge experiment, 12 were nominally differentially expressed between malaria patients in the city of Cotonou and rural village of Zinvie in Benin.

## Discussion

The core result of this study was that gene expression was significantly altered at the time of malaria diagnosis, particularly in the immunologically naïve volunteers. Although the targeted expression profiling is less comprehensive and less sensitive than the RNASeq, it suggests that there is minimal transcriptional change in peripheral blood prior to patent infection, and that individual profiles return to baseline within a few months of parasite clearance. No obvious difference in the transcriptomes of uninfected naïve and semi-immune volunteers was seen, but several hundred genes showed a stronger response in the naïve individuals. We cannot however conclude that prior immune exposure is the only reason for this difference as other lifestyle factors that distinguish the inland city of Cali from the oceanside town of Buenaventura, (where there is likely a larger proportion of African ancestry) may also play a role. However, the data is strongly suggestive of a long-term modulation of the malaria immune response involving multiple molecular pathways.

Some studies have suggested that clinically immune individuals infected with *P*. *vivax* show lower levels of inflammatory and regulatory cytokines, than individuals infected with *P*. *falciparum* malaria [21]. Nevertheless, the down-regulation of multiple genes related to innate immunity, inflammation, and neutrophil abundance, all correlated with Axis 5, observed here was unexpected. A large cross-sectional study of infants with malaria conducted in the West African Republic of Benin [9] documented a strong up-regulation of the same genes, although reanalysis of their data shown in Fig 7A suggests that is only true in the presence of high levels of parasitemia. Even more surprisingly, the reduction in inflammatory gene expression was stronger in the naïve than semi-immune volunteers. One possibility is that there is a transient reduction in relative neutrophil counts and inflammatory gene expression as the parasite first appears in the bloodstream, just as the lymphoid cells begin to amplify their response, and this is corrected as parasite levels increase and neutrophilia occurs a few days into the infection [22,23].

An observation that is consistent with published data is the strong induction of an interferon response in association with blood-stage malaria [24,25]. It is unclear whether this induction was stronger in Cali or Buenaventura, since a couple of the Cali volunteers had unusually high baseline interferon-related gene expression captured by Axis 7. It does appear that a few of the semi-immune individuals did not mount an interferon response, consistent with the absence of overt clinical symptoms and implying that their immunological memory was able to deal with at least the early stage of infection without mounting the kind of major immunological response observed in the naïve volunteers. This in turn implies that the presence of blood stage parasites alone is not the only determinant of whether or not an individual mounts an interferon response. The overall cytokine profile shifts reported in Fig 5 did not correlate with the clinical profile differences, which could be explained by the host immunity level that can vary due to the acquired immunity throughout repeated exposure [26]. Presumably larger sample sizes and longitudinal profiling during disease will identify associations between gene expression and physiological response, which is also likely to involve other tissues.

On the other hand, multiple classes of gene activity do seem to be differentially activated between naïve and semi-immune volunteers. These include various signal transduction molecules, genes related to macrophage activity, and other cellular processes that are known to influence immune responsiveness including lipid synthesis and lysosomal function concordant with Portugal et al. [27] who suggest that as children develop exposure-dependent immunity to *P*. *falciparum*, the responses reduce pathogenic inflammation and boost anti-parasite mechanisms. The aforementioned study in Benin again provides a potential comparison, since it included the contrast between children in the city of Cotonou with the rural village of Zinvié. Differences in human peripheral blood gene expression according to lifestyle are prevalent [28], but it is nevertheless interesting that, of the 32genes showing a significant interaction effect between timepoint and population in our challenge experiment, 12 were nominally differentially expressed between malaria patients from the two locations in Benin, compared with no more than three expected. Fig 7B shows that Axis 1 (related to T-cell signaling) is down-regulated with high parasitemia, and consistently reduced in the village of Zinvié. This Axis was not affected in our study, but collectively these observations of context-dependent alterations in gene expression provide further evidence that immune history is an important mediator of the differential clinical profiles observed among individuals.

There is also considerable interest in the use of gene expression profiling to identify genes that may mediate robust vaccine responses. Recent study reports on influenza and yellow fever have highlighted individual genes that are required for vaccine effectiveness, but have also suggested that baseline profiles of immune cell types may provide better predictors of antibody production [8,29]. Various properties of *Plasmodium* suggest that this organism may present a more difficult scenario for dissecting the molecular basis of vaccine responses, but we consider the results reported here to be an encouraging baseline establishing that differential responses to a malaria challenge can be detected by gene expression profiling. It will be interesting to see whether pre-immune exposure influences the molecular basis of vaccination with irradiated sporozoites in the next phase of this study.

This study shows that differential gene expression is particularly strong in naïve volunteers in comparison to semi-immune individuals at the time of malaria diagnosis. One way to interpret this result is that it provides a molecular signature of tolerance of, as opposed to resistance to, the pathogen [30]. In the presence of chronic exposure, the host immune system moves toward an equilibrium where pathogen is tolerated by mounting a measured immune response, without requiring complete sterile immunity that would likely have a greater physiological impact on the infected individual. This in turn implies that gene expression profiling of lymphocytes can be used to identify the type and duration of the immune signals that may be biomarkers for vaccine immunogenicity, and to establish how semi-immune exposure modifies their activation.

## Supporting Information

S1 Table Transformed Fluidigm data. (XLS)

S2 Table Quality control metrics for RNASeq. (XLS)

S3 Table SNM normalized RNASeq data. (XLS)

S4 Table List of genes correlated with each BIT Axis. (XLS)

S5 Table Blood informative transcript (BIT) axes values for Fluidigm (A) and RNASeq (B) data. (XLS)

S6 Table Magnitude and significance of differentially expressed genes. (XLS)
